# Genetics and pathogenesis of idiopathic scoliosis

**DOI:** 10.1186/s13013-016-0105-8

**Published:** 2016-11-28

**Authors:** A. Grauers, E. Einarsdottir, P. Gerdhem

**Affiliations:** 1 Department of Orthopaedics, Sundsvall and Härnösand County Hospital, Sundsvall, Sweden; 2 Department of Clinical Science, Intervention and Technology (CLINTEC), Karolinska Institutet, SE-141 86 Stockholm, Sweden; 3 Molecular Neurology Research Program, University of Helsinki and Folkhälsan Institute of Genetics, Helsinki, Finland; 4 Department of Biosciences and Nutrition, Karolinska Institutet, SE-141 83 Huddinge, Sweden; 5 Department of Orthopaedics, Karolinska University Hospital, SE-141 86 Stockholm, Sweden

**Keywords:** Idiopathic scoliosis, Genetics, Pathogenesis, Heredity

## Abstract

Idiopathic scoliosis (IS), the most common spinal deformity, affects otherwise healthy children and adolescents during growth. The aetiology is still unknown, although genetic factors are believed to be important. The present review corroborates the understanding of IS as a complex disease with a polygenic background. Presumably IS can be due to a spectrum of genetic risk variants, ranging from very rare or even private to very common. The most promising candidate genes are highlighted.

## Background

Idiopathic scoliosis (IS), the most common form of spinal deformity, affects otherwise healthy children and adolescents during growth. It usually presents as a rib hump visible at forward bending, together with unleveled shoulders and an asymmetrical waist. According to Cobb [1], the diagnosis is confirmed by a standing spinal radiograph showing a lateral curvature of the spine exceeding 10°. The aetiology is still unknown, although hereditary and genetic factors are believed to be important.

A major concern in IS is the absence of reliable means by which to predict risk of progression, leading to frequent follow-ups, radiographs, and potentially unnecessary brace treatments. A further understanding of the pathogenesis and genetics in IS might help in identifying at-risk individuals, leading to an earlier diagnosis and possibly better preventive and therapeutic options. The aim of the present review is to give an overview of current research in the area; the literature search strategy is outlined in Table 1.

**Table 1:** Literature search strategy

Literature search strategy	We searched Pubmed using the following search terms: idiopathic AND ‘scoliosis’ AND, ‘etiology’, OR ‘heredity’ OR ‘genetics’ OR ‘pathogenesis’.
Selection criteria	The reference lists of articles and reviews identified by this search strategy were scrutinized and references were included when judged relevant.

## Clinical presentation

The prevalence of IS is approximately 2–3% worldwide [2–4]. Most individuals have small curvatures, girls and boys being equally affected. Approximately 10% progress to a moderate or severe curve [3, 4]. Among those with severe curves, the percentage of boys is less than 10% [5]. The clinical manifestation or phenotype of IS is highly variable: the apex of the major curve may be thoracic, thoracolumbar or lumbar and the convexity may be either left or right-sided, with compensatory curvatures above and below. The most common form is a right thoracic convexity with a compensatory left lumbar convexity. A left thoracic convexity is uncommon and more often associated with asymptomatic neural axis abnormalities [6].

A young age at onset, large curvature at presentation, a thoracic curve pattern, and skeletal immaturity increase the likelihood of progression [7, 8]. Thoracic curves in the skeletally immature individual have the highest risk of progression, 58–100% [8–10]. When the individual stops growing, the risk of progression diminishes. At skeletal maturity, curves of less than 30° carry a very small risk of progression. In contrast, curves that reach 50° tend to continue to progress throughout adulthood, at a rate of approximately 1° per year [9].

## Aetiology and pathogenesis

The pathogenesis of scoliosis is poorly understood. It is not unreasonable to believe that an existing deformity might produce an asymmetrical loading of the growing spine, which in turn would cause asymmetrical growth of the vertebrae. But how does it start? And why is it progressive in some but not in others? Biomechanical, neural, metabolic and hormonal changes have been reported in IS but it is difficult to say whether these are primary or secondary to the deformity. Various theories based on these findings have been suggested, some of these are listed below.

In 1959, Thillard [11] discovered that pinealectomised chickens developed scoliosis. This was repeated in bi-pedalised rats and a deficiency of melatonin was suggested to be causative of IS [12, 13]. Further studies, however, showed that adolescent IS patients had normal melatonin levels [14], and pinealectomised monkeys did not develop scoliosis [15]. Instead, a melatonin-signaling pathway dysfunction affecting only certain cell types, notably osteoblasts, was suggested [16, 17]. Calmodulin, a calcium-binding receptor protein, regulates contractile properties in platelets and muscles, and interacts with melatonin. Increased levels of calmodulin in platelets and an asymmetrical distribution of calmodulin in paraspinal muscles compared to healthy controls have been described in IS patients [18, 19].

Dickson et al. [20] showed that vertebral bodies were wedged in the sagittal plane in IS patients, causing an apical lordosis in thoracic curvatures. They suggested that this lordosis, in a region that is normally kyphotic, created a rotation of the spine and, secondarily, a lateral spinal curvature. On MRI scans of IS patients it has been shown that the spinal cord is shorter in relation to the vertebral column [21], that there is an increased prevalence of cerebellar tonsillar ectopia [22], as well as an uncoordinated growth of the vertebral bodies in relation to the dorsal elements [23], compared to controls. This has led to theories postulating a relative anterior spinal overgrowth (RASO) or an uncoupled neuro-osseus growth as a cause of IS [24].

As previously described, the risk of curve progression in IS is related to skeletal immaturity. It has also been shown that girls with adolescent IS are taller [25–27] and have a higher growth velocity during puberty compared to healthy controls [28–30]. Subsequently, bone mineral density, growth, and sex hormones have been studied in the pathogenesis of IS. Cheung et al. [27] showed that adolescent girls with IS had lower bone mineral density than healthy controls, and a higher bone turnover rate. In the same cohort, Hung et al. [31] found that low bone mineral density in the femoral neck was associated with curve progression.

Gerdhem et al. [32] showed a decreased level of COMP, cartilage oligomeric matrix protein, in serum in IS patients compared to controls. COMP has previously been associated with growth velocity in juvenile rheumatoid arthritis [33]. In addition, raised levels of growth hormone (GH) and insulin-like growth factor 1 (IGF-1) have been associated with IS [34, 35], as well as lower circulating levels of leptin, the “satiety” hormone [36]. Oestrogen levels have also been studied, but with inconclusive results [37].

## Heredity and genetics

It has long been known that hereditary factors play a role in the aetiology of IS. Inheritance of scoliosis in five generations was described by Garland in 1934 [38].

In 1968, Wynne-Davis [39] and in 1973 Riseborough and Wynne-Davis [40] reported on the familial occurrence of IS in British and American cohorts. The proportion of study participants having a relative with IS was 27 and 26%, respectively. The prevalence of scoliosis among first-degree relatives was 7% and 16%, which is significantly higher than in the general population. Tang et al. [41] showed a sibling recurrence risk of scoliosis of 18% in a Chinese cohort of 415 female adolescent IS patients with Cobb > 20°. In a cohort of 1,463 individuals with IS, we found that among those treated with a brace or surgery for scoliosis, 53% reported one or more relatives with scoliosis, compared to 46% of the untreated, pointing towards a slightly higher risk of treatment in the presence of a family history of scoliosis [42].

In addition, several twin studies have reported a higher concordance of IS (meaning that both twins have the disorder) in monozygotic compared to dizygotic twin pairs, indicating a genetic influence [43–45]. We have analysed self-reported data on scoliosis in twins (*n* = 64,578) in the population-based Swedish Twin Registry and estimated the relative importance of genetic effects on the phenotypic variance (i.e. the heritability) to be 38% [46].

As a consequence there has been a vast amount of genetic research on IS. A short description of different approaches in genetic research as well as a summary of the findings on IS are given below.

## Genetic approaches

**Fig. 1:**
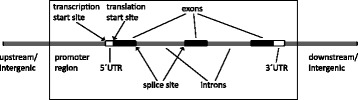
Gene anatomy. Intergenic regions: areas between genes. Transcription start site: starting point for the RNA transcription (from DNA template). Translation start site: starting point for protein translation (from mRNA template). Exons: retained in mRNA, basis for protein translation. Promoter region: regulatory region important for initiation of transcription. 5′UTR and 3′UTR: non-coding start and endpoint of mRNA. Splice site: sequence that guides splicing of exonic RNA. Introns: sequence removed during splicing. Artwork: Elísabet Einarsdóttir

Sequencing allows us to determine the nucleotide sequence of a DNA strand, and thus potentially discover new mutations or genetic variants. Sequencing a whole genome, however, yields immense amounts of data and requires large amounts of downstream bioinformatic analysis. Severe phenotypes could be assumed to be due to mutations in the protein-coding rather than the non-coding areas of the genome. One option could then be to sequence only the protein-coding regions, the so-called exome, which constitutes approximately 1% of the genome, Fig. 1.

**Fig. 2:**
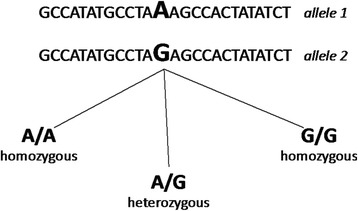
Example of a single nucleotide variation (SNV). Upper part: DNA sequence illustrating two possible alleles at a specific point in the genome. This type of variation is called single-nucleotide variation (SNV). Lower part: the resulting three possible genotypes. Artwork: Elísabet Einarsdóttir

Genotyping, in contrast to sequencing, depends on the knowledge of known variants – for example, single-nucleotide variants (SNV), with known positions in the genome, Fig. 2. An assay is set up to test for the specific variant(s), meaning that one tests which of the possible alleles or versions of the variation the individual has at that specific point. Compared to sequencing, this is a very efficient method of finding out if a certain known variation is associated with a disease. Genotyping can be used in both genome-wide and candidate-gene approaches.

In genome-wide approaches, millions of variants throughout the genome are assayed simultaneously. This approach is useful when one has no prior hypothesis of what region or gene might be involved in the disease. However, it is rather expensive and yields massive amounts of data, and the criteria for significance of the data are often quite stringent due to a need for multiple testing correction. If there is a solid hypothesis of what gene(s) might be involved in the disorder, one can instead elect to test variations in this specific area only – a so called candidate-gene approach. The latter approach is more straightforward and allows for a more detailed analysis of a candidate gene, but it is highly dependent on the initial assumptions of the study design. It would also not be helpful for discovering completely new and previously unsuspected disease mechanisms.

Genotyping is used in both association and linkage studies. In association studies one compares the frequency of specific versions/forms/alleles of genetic variants in cases and controls. Association studies can establish whether common known genetic variants are associated with a disorder, even if they only have a weak effect on the phenotype or low penetrance. The existence of a variant in an individual is usually not diagnostic for the disease, but rather indicates an (often subtly) increased disease susceptibility. Even if a specific variant increases the person’s susceptibility to a disease by only 5%, this can be a very important modulator of disease risk in the population if the variant is common.

Linkage studies, on the other hand, analyse the co-segregation of a phenotype and a mutation in families. Both large and small families can be used. DNA markers or SNVs are analysed either at a certain point of interest or genome-wide. It is then possible to link a region of the genome with the phenotype. The advantage of linkage studies is that one does not need to know what one is looking for in advance, and a study of multiple families could yield a linkage signal in common even if the disease-causing mutations underlying the linkage signal differed between families. A limitation is that a strong correlation between the phenotype and genotype is needed (a high penetrance), making linkage a more powerful approach for phenotypes of more classical Mendelian inheritance (e.g. recessive or dominant). This type of study can have diagnostic value for members of families carrying a rare, monogenic disease, but the relevance of such findings for the general population is unclear.

## Summary of genetic findings in idiopathic scoliosis

### Exome sequencing

Baschal et al. [47] sequenced the exomes of three affected individuals in a multigenerational family with dominant Mendelian inheritance of IS. They identified a rare missense variant in *HSPG2*, coding for an extracellular matrix protein, also known as perlecan. They further sequenced exons of *HSPG* in 100 independent IS patients and found 21 other potentially damaging variants in *HSPG*. Buchan et al. [48] exome-sequenced a cohort of 91 individuals with severe IS and compared the results with 337 controls. Using a gene burden analysis, they found that variants within the *fibrillin 1* and *2* genes were associated with IS. Mutations in *fibrillin 1* are known to be associated with Marfan syndrome, in which a high proportion of patients develops scoliosis.

Haller et al. [49] exome-sequenced 391 severe adolescent IS cases and 843 controls. Using a pathway burden analysis, they found that variants in extracellular matrix genes, especially in musculoskeletal collagen, were significantly enriched in adolescent IS cases compared to controls. Individual genotyping in 919 cases revealed a highly significant association with *COL11A2*, which encodes a fibrillar collagen.

### Genome-wide association studies (GWAS)

Several genome-wide association studies in adolescent IS have been reported. The most interesting findings are shown in Table 2.

**Table 2:** The most important single nucleotide variants (SNV) found to be associated with idiopathic scoliosis in GWAS

SNV name	Gene	RAF cases	RAF controls	P-value	OR(95%CI)	Reference	Replicated
rs11190870	*LBX1* downstream	0.67	0.57	1.2 × 10^−19^	1.6(1.4–1.7)	Takahashi et al. 2011 [50]	Londono et al. 2014 [51], Gao et al. 2013 [52], Grauers et al. 2015 [53]
rs657507	*GPR126* intron	0.32	0.28	1.6 × 10^−4^	1.2(1.1–1.4)	Kou et al. 2013 [61]	Xu et al.2015 [62] Grauers et al. 2015 [53]
rs12946942	Intergenic	0.26	0.21	4.0 × 10^−8^	2.0(1.6–2.7)	Miyake et al. 2013 [64]	Grauers et al. 2015 [53]
rs3904778	*BNC2*	-	-	2.5 × 10^−13^	1.2(1.2–1.3)	Ogura et al.2015 [63]	-
rs6137473	*PAX1*	-	-	2.2 × 10^−10^	1.3(1.2–1.4)	Sharma et al. 2015 [68]	-

*RAF* Risk allele frequency, *OR* Odds ratio, *CI* Confidence interval

In 2011, Takahashi et al. [50] performed a large GWAS in a Japanese population and found an association with a variant downstream of the *LBX1* (ladybird homeobox 1) gene. This finding was later replicated in both Chinese Han and Caucasian populations, as well as by us in a Scandinavian population [51–53]. The function of *LBX1* is largely unknown but it is expressed in dorsal spinal neurons and hindbrain, muscle precursor cells, and certain cardiac crest cells [54–58]. Fernandez-jaen et al. [59] reported a clinical case involving scoliosis and myopathy due to a microduplication in the chromosomal region of 10q24.31 affecting exclusively *LBX1*. Recently, Guo et al. [60] found that the associated variant facilitates transcription of *LBX1* and that overexpression of *LBX1* causes body axis deformation in zebrafish.

Kou et al. [61] found an association of *GPR126* (G-protein coupled receptor 126) to IS in a GWAS in populations of Japanese, Han Chinese and European ancestry. This finding has been replicated in a small Chinese candidate gene study [62]. A knockdown of *GPR126* in zebrafish caused delayed ossification of the developing spine [61].

Through an extended GWAS and replication studies using independent Japanese and Chinese populations, the same group found an association of a variant in the *BNC2* (basonuclin-2) gene, which encodes a zinc finger transcription factor. *BNC2* overexpression induced body curvature in developing zebrafish in a gene-dosage-dependent manner [63].

In a GWAS of severe cases of adolescent IS in the Japanese and Chinese populations, Miyake et al. [64] found association to the variant rs12946942 on chromosome 17q24.3 near the genes *SOX9* and *KCNJ2*. Mutations within these genes are associated with campomelic dysplasia and Andersen-Tawil syndrome, both demonstrating a scoliotic phenotype in addition to other symptoms.

Ward et al. [65] identified 53 variants associated with curve progression of adolescent IS in a GWAS that is not yet published and validated them in a Caucasian cohort. They suggested that these variants could be useful for predicting progression of scoliosis. However, the association of these variants to progression of scoliosis has not been replicated in either a Japanese or a French-Canadian cohort [66, 67].

By performing a GWAS of 3,102 individuals, Sharma et al. [68] identified significant association of a locus distal to *PAX1* to IS in female patients. *PAX1* codes for Paired box 1, a transcription factor involved in spine development. The associated locus has showed enhancer activity in zebrafish somitic muscle and spinal cord.

### Candidate gene association studies

Inspired by speculations on the pathogenesis of IS, various candidate genes related to bone metabolism, connective tissue, the melatonin-signaling pathway, growth and sex hormones have been investigated [69]. Most of these associations have not been replicated in later larger studies [69–74]. Recent candidate gene studies have shown an association between *IL*-*17RC* (interleukin 17 receptor C), *TGFB1* (transforming growth factor beta 1), genes correlated with peak height velocity during puberty, *DOT1L* and *C17orf67*, and IS [75–77].

### Linkage and inheritance models

Several genome-wide linkage studies have been performed on IS families [69]. Both autosomal dominant, X-linked dominant and autosomal recessive models of inheritance have been suggested and different chromosomal regions have shown linkage in different subsets of families. Gao et al. [78] suggested linkage to the chromosomal region 8q12, and fine-mapping of this area revealed the *CDH7* gene. Mutations in *CDH7* are responsible for the CHARGE syndrome, in which a high percentage of patients develop scoliosis. This finding could, however, not be replicated in another subset of families [79]. Edery et al. [80] suggested linkage to the regions 3q12.1 and 5q13.3 in a multigenerational family. In a follow-up using exome sequencing of three affected members of this family, a novel rare missense variant in *POC5*, a centripolar protein, was discovered. This variant caused spine deformity in a zebrafish model [81].

### Other approaches

Fendri et al. [82] compared mRNA expression in primary osteoblasts from vertebrae in adolescent IS patients and healthy controls and found 145 genes differentially expressed in osteoblasts. The most significant changes in expression levels were observed in homeobox genes, as well as in *ZIC2, FAM101A, COMP* and *PITX1*. These genes interact in the biological pathways of bone development, particularly in the differentiation of skeletal elements and the structural integrity of the vertebrae [82]. Buchan et al. [83] reported rare copy number variations (CNVs) in a cohort of 143 IS patients. These genes have not previously been investigated in IS.

### Drawbacks

Genetic studies are limited by their design. A genetic approach focused on finding common variants (GWAS) will not reveal rare variants, on the other hand linkage studies of a family may identify disease causing variants in that specific family but these findings might not be applicable in the majority of patients. As the pathogenetic mechanism(s) of idiopathic scoliosis is/are not well known, a number of different genetic approaches has been used and the various genetic findings reported reflect the chosen methods. However, instead of being contradictory one may interpret the results (of well conducted studies) as small pieces in a large genetic puzzle. The limitations of the present review include the possibility that we have failed to analyse some studies on the subject and that the conclusion is a result of our understanding of the field rather than evidence.

## Conclusion

The present review corroborates the understanding of IS as a complex disease with a polygenic background. IS can presumably be due to a spectrum of genetic risk variants, ranging from very rare or even private to very common in the general population. The risk effect of the variants could also range from quite severe to very mild and even undetectable in practice. The most promising common gene variant discovered so far is rs11190870 downstream of the *LBX1* gene. This variant is shown to increase disease susceptibility in several populations and a plausible effect mechanism has been presented [50–53, 60]. An intronic variant within *GPR126* and the intergenic variant rs12946942 have also been replicated in different populations, however the precise functional effect of these variants has yet to be elucidated [61, 64]. Two other common variants of interest have recently been found to be associated with IS, but have yet to be replicated (Table 2) [63, 68]. New methodologies such as exome-sequencing have made it possible to identify rare variants associated with idiopathic scoliosis [47–49, 81]. The importance of these findings in the general idiopathic scoliosis population, however, remains to be seen.

Future strategies for revealing the pathogenic mechanism underlying scoliosis might be studying families with monogenic IS in order to find a causative mutation. A possible finding will not explain the specific genetic background in the general IS population but might reveal biological pathways that are important in all or most forms of IS. In addition there are several genetic syndromes, of both known and unknown causes, in which scoliosis is part of the phenotype. Further studies of these disorders could add information to the pathogenic mechanism of scoliosis development. Yet another possibility is international collaboration, collecting even larger cohorts of IS patients. A large sample size would better enable us to find association with rare variants.

## References

[1] Cobb J (1948). Technique for study of scoliosis. W P Blount AAoOS ed AAOS Instructional Course Lectures.

[2] Rogala EJ, Drummond DS, Gurr J (1978). Scoliosis: incidence and natural history. A prospective epidemiological study. J Bone Joint Surg Am.

[3] Willner S, Uden A (1982). A prospective prevalence study of scoliosis in Southern Sweden. Acta Orthop Scand.

[4] Luk KD, Lee CF, Cheung KM, Cheng JC, Ng BK, Lam TP (2010). Clinical effectiveness of school screening for adolescent idiopathic scoliosis: a large population-based retrospective cohort study. Spine.

[5] Montgomery F, Willner S (1988). The natural history of idiopathic scoliosis. A study of the incidence of treatment. Spine.

[6] Wu L, Qiu Y, Wang B, Zhu ZZ, Ma WW (2010). The left thoracic curve pattern: a strong predictor for neural axis abnormalities in patients with "idiopathic" scoliosis. Spine.

[7] Bunnell WP (1988). The natural history of idiopathic scoliosis. Clin Orthop Relat Res.

[8] Lonstein JE, Carlson JM (1984). The prediction of curve progression in untreated idiopathic scoliosis during growth. J Bone Joint Surg Am.

[9] Weinstein SL, Zavala DC, Ponseti IV (1981). Idiopathic scoliosis: long-term follow-up and prognosis in untreated patients. J Bone Joint Surg Am.

[10] Weinstein SL, Ponseti IV (1983). Curve progression in idiopathic scoliosis. J Bone Joint Surg Am.

[11] Thillard MJ (1959). Vertebral column deformities following epiphysectomy in the chick. C R Hebd Seances Acad Sci.

[12] Machida M, Murai I, Miyashita Y, Dubousset J, Yamada T, Kimura J (1999). Pathogenesis of idiopathic scoliosis. Experimental study in rats. Spine.

[13] Machida M, Dubousset J, Imamura Y, Iwaya T, Yamada T, Kimura J (1995). Role of melatonin deficiency in the development of scoliosis in pinealectomised chickens. J Bone Joint Surg Br.

[14] Fagan AB, Kennaway DJ, Sutherland AD (1998). Total 24-hour melatonin secretion in adolescent idiopathic scoliosis. A case–control study. Spine.

[15] Cheung KM, Wang T, Poon AM, Carl A, Tranmer B, Hu Y (2005). The effect of pinealectomy on scoliosis development in young nonhuman primates. Spine.

[16] Moreau A, Wang DS, Forget S, Azeddine B, Angeloni D, Fraschini F (2004). Melatonin signaling dysfunction in adolescent idiopathic scoliosis. Spine.

[17] Wang WW, Man GC, Wong JH, Ng TB, Lee KM, Ng BK (2014). Abnormal response of the proliferation and differentiation of growth plate chondrocytes to melatonin in adolescent idiopathic scoliosis. Int J Mol Sci.

[18] Lowe T, Lawellin D, Smith D, Price C, Haher T, Merola A (2002). Platelet calmodulin levels in adolescent idiopathic scoliosis: do the levels correlate with curve progression and severity?. Spine.

[19] Acaroglu E, Akel I, Alanay A, Yazici M, Marcucio R (2009). Comparison of the melatonin and calmodulin in paravertebral muscle and platelets of patients with or without adolescent idiopathic scoliosis. Spine.

[20] Dickson RA, Lawton JO, Archer IA, Butt WP (1984). The pathogenesis of idiopathic scoliosis. Biplanar spinal asymmetry. J Bone Joint Surg Br.

[21] Chu WC, Lam WW, Chan YL, Ng BK, Lam TP, Lee KM (2006). Relative shortening and functional tethering of spinal cord in adolescent idiopathic scoliosis?: study with multiplanar reformat magnetic resonance imaging and somatosensory evoked potential. Spine.

[22] Abul-Kasim K, Overgaard A, Karlsson MK, Ohlin A (2009). Tonsillar ectopia in idiopathic scoliosis: does it play a role in the pathogenesis and prognosis or is it only an incidental finding?. Scoliosis.

[23] Guo X, Chau WW, Chan YL, Cheng JC (2003). Relative anterior spinal overgrowth in adolescent idiopathic scoliosis. Results of disproportionate endochondral-membranous bone growth. J Bone Joint Surg Br.

[24] Chu WC, Lam WM, Ng BK, Tze-Ping L, Lee KM, Guo X (2008). Relative shortening and functional tethering of spinal cord in adolescent scoliosis - Result of asynchronous neuro-osseous growth, summary of an electronic focus group debate of the IBSE. Scoliosis.

[25] Normelli H, Sevastik J, Ljung G, Aaro S, Jonsson-Soderstrom AM (1985). Anthropometric data relating to normal and scoliotic Scandinavian girls. Spine.

[26] Siu King Cheung C, Tak Keung Lee W, Kit Tse Y, Ping Tang S, Man Lee K, Guo X (2003). Abnormal peri-pubertal anthropometric measurements and growth pattern in adolescent idiopathic scoliosis: a study of 598 patients. Spine.

[27] Cheung CS, Lee WT, Tse YK, Lee KM, Guo X, Qin L (2006). Generalized osteopenia in adolescent idiopathic scoliosis--association with abnormal pubertal growth, bone turnover, and calcium intake?. Spine.

[28] Willner S (1974). A study of growth in girls with adolescent idiopathic structural scoliosis. Clin Orthop Relat Res.

[29] Wang WJ, Hung VW, Lam TP, Ng BK, Qin L, Lee KM (2010). The association of disproportionate skeletal growth and abnormal radius dimension ratio with curve severity in adolescent idiopathic scoliosis. Eur Spine J.

[30] Chazono M, Soshi S, Kida Y, Hashimoto K, Inoue T, Nakamura Y (2012). Height velocity curves in female patients with idiopathic scoliosis. Stud Health Technol Inform.

[31] Hung VW, Qin L, Cheung CS, Lam TP, Ng BK, Tse YK (2005). Osteopenia: a new prognostic factor of curve progression in adolescent idiopathic scoliosis. J Bone Joint Surg Am.

[32] Gerdhem P, Topalis C, Grauers A, Stubendorff J, Ohlin A, Karlsson KM (2015). Serum level of cartilage oligomeric matrix protein is lower in children with idiopathic scoliosis than in non-scoliotic controls. Eur Spine J.

[33] Bjornhart B, Juul A, Nielsen S, Zak M, Svenningsen P, Muller K (2009). Cartilage oligomeric matrix protein in patients with juvenile idiopathic arthritis: relation to growth and disease activity. J Rheumatol.

[34] Sanders JO, Browne RH, Cooney TE, Finegold DN, McConnell SJ, Margraf SA (2006). Correlates of the peak height velocity in girls with idiopathic scoliosis. Spine.

[35] Willner S, Johnell O (1981). Study of biochemical and hormonal data in idiopathic scoliosis in girls. Arch Orthop Trauma Surg.

[36] Qiu Y, Sun X, Qiu X, Li W, Zhu Z, Zhu F (2007). Decreased circulating leptin level and its association with body and bone mass in girls with adolescent idiopathic scoliosis. Spine.

[37] Lombardi G, Akoume MY, Colombini A, Moreau A, Banfi G (2011). Biochemistry of adolescent idiopathic scoliosis. Adv Clin Chem.

[38] Garland HG (1934). Hereditary scoliosis. Br Med J.

[39] Wynne-Davies R (1968). Familial (idiopathic) scoliosis. A family survey. J Bone Joint Surg Br.

[40] Riseborough EJ, Wynne-Davies R (1973). A genetic survey of idiopathic scoliosis in Boston, Massachusetts. J Bone Joint Surg Am.

[41] Tang NL, Yeung HY, Hung VW, Di Liao C, Lam TP, Yeung HM (2012). Genetic epidemiology and heritability of AIS: A study of 415 Chinese female patients. J Orthop Res.

[42] Grauers A, Danielsson A, Karlsson M, Ohlin A, Gerdhem P (2013). Family history and its association to curve size and treatment in 1,463 patients with idiopathic scoliosis. Eur Spine J.

[43] Kesling KL, Reinker KA (1997). Scoliosis in twins. A meta-analysis of the literature and report of six cases. Spine.

[44] Inoue M, Minami S, Kitahara H, Otsuka Y, Nakata Y, Takaso M (1998). Idiopathic scoliosis in twins studied by DNA fingerprinting: the incidence and type of scoliosis. J Bone Joint Surg Br.

[45] Andersen MO, Thomsen K, Kyvik KO (2007). Adolescent idiopathic scoliosis in twins: a population-based survey. Spine.

[46] Grauers A, Rahman I, Gerdhem P (2012). Heritability of scoliosis. Eur Spine J.

[47] Baschal EE, Wethey CI, Swindle K, Baschal RM, Gowan K, Tang NL (2014). Exome Sequencing Identifies a Rare HSPG2 Variant Associated with Familial Idiopathic Scoliosis. G3 (Bethesda, Md).

[48] Buchan JG, Alvarado DM, Haller GE, Cruchaga C, Harms MB, Zhang T (2014). Rare variants in FBN1 and FBN2 are associated with severe adolescent idiopathic scoliosis. Hum Mol Genet.

[49] Haller G, Alvarado D, McCall K, Yang P, Cruchaga C, Harms M (2016). A polygenic burden of rare variants across extracellular matrix genes among individuals with adolescent idiopathic scoliosis. Hum Mol Genet.

[50] Takahashi Y, Kou I, Takahashi A, Johnson TA, Kono K, Kawakami N (2011). A genome-wide association study identifies common variants near LBX1 associated with adolescent idiopathic scoliosis. Nat Genet.

[51] Londono D, Kou I, Johnson TA, Sharma S, Ogura Y, Tsunoda T (2014). A meta-analysis identifies adolescent idiopathic scoliosis association with LBX1 locus in multiple ethnic groups. J Med Genet.

[52] Gao W, Peng Y, Liang G, Liang A, Ye W, Zhang L (2013). Association between common variants near LBX1 and adolescent idiopathic scoliosis replicated in the Chinese Han population. PLoS One.

[53] Grauers A, Wang J, Einarsdottir E, Simony A, Danielsson A, Akesson K (2015). Candidate gene analysis and exome sequencing confirm LBX1 as a susceptibility gene for idiopathic scoliosis. Spine J.

[54] Jagla K, Dolle P, Mattei MG, Jagla T, Schuhbaur B, Dretzen G (1995). Mouse Lbx1 and human LBX1 define a novel mammalian homeobox gene family related to the Drosophila lady bird genes. Mech Dev.

[55] Schafer K, Braun T (1999). Early specification of limb muscle precursor cells by the homeobox gene Lbx1h. Nat Genet.

[56] Gross MK, Dottori M, Goulding M (2002). Lbx1 specifies somatosensory association interneurons in the dorsal spinal cord. Neuron.

[57] Schafer K, Neuhaus P, Kruse J, Braun T (2003). The homeobox gene Lbx1 specifies a subpopulation of cardiac neural crest necessary for normal heart development. Circ Res.

[58] Cheng L, Samad OA, Xu Y, Mizuguchi R, Luo P, Shirasawa S (2005). Lbx1 and Tlx3 are opposing switches in determining GABAergic versus glutamatergic transmitter phenotypes. Nat Neurosci.

[59] Fernandez-Jaen A, Suela J, Fernandez-Mayoralas DM, Fernandez-Perrone AL, Wotton KR, Dietrich S (2014). Microduplication 10q24.31 in a Spanish girl with scoliosis and myopathy: the critical role of LBX. Am J Med Genet A.

[60] Guo L, Yamashita H, Kou I, Takimoto A, Meguro-Horike M, Horike S (2016). Functional Investigation of a Non-coding Variant Associated with Adolescent Idiopathic Scoliosis in Zebrafish: Elevated Expression of the Ladybird Homeobox Gene Causes Body Axis Deformation. PLoS Genet.

[61] Kou I, Takahashi Y, Johnson TA, Takahashi A, Guo L, Dai J (2013). Genetic variants in GPR126 are associated with adolescent idiopathic scoliosis. Nat Genet.

[62] Xu JF, Yang GH, Pan XH, Zhang SJ, Zhao C, Qiu BS (2015). Association of GPR126 gene polymorphism with adolescent idiopathic scoliosis in Chinese populations. Genomics.

[63] Ogura Y, Kou I, Miura S, Takahashi A, Xu L, Takeda K (2015). A Functional SNP in BNC2 Is Associated with Adolescent Idiopathic Scoliosis. Am J Hum Genet.

[64] Miyake A, Kou I, Takahashi Y, Johnson TA, Ogura Y, Dai J (2013). Identification of a susceptibility locus for severe adolescent idiopathic scoliosis on chromosome 17q24.3. PLoS One.

[65] Ward K, Ogilvie JW, Singleton MV, Chettier R, Engler G, Nelson LM (2010). Validation of DNA-based prognostic testing to predict spinal curve progression in adolescent idiopathic scoliosis. Spine.

[66] Ogura Y, Takahashi Y, Kou I, Nakajima M, Kono K, Kawakami N (2013). A replication study for association of 53 single nucleotide polymorphisms in a scoliosis prognostic test with progression of adolescent idiopathic scoliosis in Japanese. Spine.

[67] Tang QL, Julien C, Eveleigh R, Bourque G, Franco A, Labelle H, et al. A Replication Study for Association of 53 Single Nucleotide Polymorphisms in ScoliScore Test with Adolescent Idiopathic Scoliosis in French-Canadian Population. Spine. 2015;40(8):537-43. 10.1097/BRS.0000000000000807 25646748

[68] Sharma S, Londono D, Eckalbar WL, Gao X, Zhang D, Mauldin K (2015). A PAX1 enhancer locus is associated with susceptibility to idiopathic scoliosis in females. Nat Commun.

[69] Gorman KF, Julien C, Moreau A (2012). The genetic epidemiology of idiopathic scoliosis. Eur Spine J.

[70] Nelson LM, Ward K, Ogilvie JW (2011). Genetic variants in melatonin synthesis and signaling pathway are not associated with adolescent idiopathic scoliosis. Spine.

[71] Ogura Y, Takahashi Y, Kou I, Nakajima M, Kono K, Kawakami N (2013). A replication study for association of 5 single nucleotide polymorphisms with curve progression of adolescent idiopathic scoliosis in Japanese patients. Spine.

[72] Yang M, Li C, Li M (2014). The estrogen receptor alpha gene (XbaI, PvuII) polymorphisms and susceptibility to idiopathic scoliosis: a meta-analysis. J Orthop Sci.

[73] Chen S, Zhao L, Roffey DM, Phan P, Wai EK (2014). Association between the ESR1–351A > G single nucleotide polymorphism (rs9340799) and adolescent idiopathic scoliosis: a systematic review and meta-analysis. Eur Spine J.

[74] Zhang H, Zhao S, Zhao Z, Tang L, Guo Q, Liu S (2014). The association of rs1149048 polymorphism in matrilin-1(MATN1) gene with adolescent idiopathic scoliosis susceptibility: a meta-analysis. Mol Biol Rep.

[75] Zhou S, Qiu XS, Zhu ZZ, Wu WF, Liu Z, Qiu Y (2012). A single-nucleotide polymorphism rs708567 in the IL-17RC gene is associated with a susceptibility to and the curve severity of adolescent idiopathic scoliosis in a Chinese Han population: a case–control study. BMC Musculoskelet Disord.

[76] Mao S, Xu L, Zhu Z, Qian B, Qiao J, Yi L (2013). Association between genetic determinants of peak height velocity during puberty and predisposition to adolescent idiopathic scoliosis. Spine (Phila Pa 1976).

[77] Ryzhkov II, Borzilov EE, Churnosov MI, Ataman AV, Dedkov AA, Polonikov AV (2013). Transforming Growth Factor Beta 1 is a Novel Susceptibility Gene for Adolescent Idiopathic Scoliosis. Spine.

[78] Gao X, Gordon D, Zhang D, Browne R, Helms C, Gillum J (2007). CHD7 gene polymorphisms are associated with susceptibility to idiopathic scoliosis. Am J Hum Genet.

[79] Tilley MK, Justice CM, Swindle K, Marosy B, Wilson AF, Miller NH (2013). CHD7 gene polymorphisms and familial idiopathic scoliosis. Spine.

[80] Edery P, Margaritte-Jeannin P, Biot B, Labalme A, Bernard JC, Chastang J (2011). New disease gene location and high genetic heterogeneity in idiopathic scoliosis. Eur J Hum Genet.

[81] Patten SA, Margaritte-Jeannin P, Bernard JC, Alix E, Labalme A, Besson A, et al. Functional variants of POC5 identified in patients with idiopathic scoliosis. J Clin Invest. 2015;125(3):1124-8. 10.1172/JCI77262 PMC4362221 25642776

[82] Fendri K, Patten SA, Kaufman GN, Zaouter C, Parent S, Grimard G (2013). Microarray expression profiling identifies genes with altered expression in Adolescent Idiopathic Scoliosis. Eur Spine J.

[83] Buchan JG, Alvarado DM, Haller G, Aferol H, Miller NH, Dobbs MB (2014). Are copy number variants associated with adolescent idiopathic scoliosis?. Clin Orthop Relat Res.

